# Morbidité et mortalité des nouveau-nés hospitalisés sur 10 années à la Clinique El Fateh-Suka (Ouagadougou, Burkina Faso)

**DOI:** 10.11604/pamj.2013.14.153.2022

**Published:** 2013-04-20

**Authors:** Kisito Nagalo, Fousséni Dao, François Housséini Tall, Diarra Yé

**Affiliations:** 1 Service de pédiatrie, Clinique El Fateh-Suka, Ouagadougou, Burkina Faso; 2 UFR/SDS Université de Ouagadougou, Burkina Faso; 3 Service de pédiatrie CHU Pédiatrique Charles de Gaulle, Ouagadougou, Burkina Faso

**Keywords:** Nouveau-né, soins périnatals, soins prénatals, mortalité néonatale, Newborn, perinatal, prenatal care, neonatal mortality

## Abstract

**Introduction:**

La mortalité néonatale demeure un problème majeur de santé publique dans les pays en développement. Notre étude avait pour but de déterminer la morbidité et la mortalité des nouveau-nés à Ouagadougou, Burkina Faso.

**Méthodes:**

Une étude rétrospective sur 10 années a permis d'inclure tous les nouveau-nés admis dans l'Unité de Néonatologie de la Clinique El Fateh-Suka.

**Résultats:**

Au total, 697 nouveau-nés étaient hospitalisés sur la période d'étude. Les principaux diagnostics étaient les infections néonatales (23.5%), les anomalies liées à la durée de la gestation et à la croissance du fætus (17.9%) et le paludisme congénital (15.1%). Les 91 (13.1%) décès étaient dus aux anomalies liées à la durée de la grossesse et à la croissance du fætus (46.1%), à l'hypoxie intra-utérine et à l'asphyxie obstétricale (20,9%) et aux infections néonatales (17.6%). Ces décès survenaient dans 81.3% dans les 72 heures, dans 93.4% des cas dans la première semaine d'hospitalisation. Le facteur de risque associé à ces décès était la voie basse d'accouchement (p = 0.02).

**Conclusion:**

Cette étude a identifié des pathologies évitables déjà décrites comme les principales causes d'hospitalisations et de décès néonatals. La voie basse d'accouchement était le facteur de risque associé à ces décès, ce qui n'avait pas encore été rapporté. Les efforts pour améliorer la qualité des services de soins périnatals doivent être intensifiés afin de réduire la mortalité néonatale dans les pays en développement.

## Introduction

Les 28 premiers jours de vie d′un enfant sont une période à haut risque d′agressions diverses qui peuvent entraîner son décès ou affecter durablement son développement et le reste de sa vie. Sur les 130 millions de naissances annuelles dans le monde, quatre millions de nouveau-nés meurent avant un mois dans 99% dans les pays pauvres pour des causes dont la plupart sont connues [1, 2] et en grande partie évitables ou accessibles à un traitement [3, 4]. Ainsi, bien plus qu′un problème de santé publique, la mortalité néonatale constitue un véritable problème de développement mondial. Parce que les nouveau-nés constituent jusqu′à 40% des décès d′enfants de moins de 5 ans [2], l′accélération de la réduction de la mortalité néonatale est impérative si l′on veut atteindre la cible 4 des Objectifs du Millénaire pour le Développement (OMD) des Nations Unies [5]. Malheureusement, la baisse du taux de mortalité néonatale ne montre pas de progrès significatif aussi bien au Burkina Faso que dans la plupart des pays au sud du Sahara où il excède 40 pour 1000 avec une baisse moyenne de moins de 1% par an [6]. Le but de notre étude était de déterminer la morbidité et la mortalité chez les nouveau-nés hospitalisés dans l′Unité de Néonatologie (UN) de la Clinique El Fateh-Suka (CFS) de Ouagadougou, Burkina Faso.

## Méthodes

### Cadre d′étude

La CFS, lieu de l'étude, est un hôpital privé situé à Ouagadougou qui possède une UN avec huit incubateurs et neuf lits. La période d′étude correspond à ses dix premières années de fonctionnement.

Une étude rétrospective sur dossiers médicaux concernant la période du 15 juin 1999 au 14 juin 2009, soit 10 années, a permis d′inclure tous les nouveau-nés hospitalisés durant la période d′étude. Ceux-ci provenaient de la maternité de l′hôpital, des autres centres de santé de la ville ou du domicile. L′étude des dossiers cliniques a permis de recueillir sur une fiche individuelle standardisée les renseignements sur les antécédents médico-obstétricaux maternels, l'accouchement, l'examen clinique, le(s) diagnostic(s) de sortie, le mode de sortie des nouveau-nés. Les données étaient saisies sur micro-ordinateur à l′aide du logiciel Epi-info 6.4 puis exportées sur SPSS 16.0 et l′analyse était faite à l′aide des deux logiciels. Pour les aspects descriptifs de l'analyse, les distributions des fréquences ont été générées pour toutes les variables. Celles qui étaient peu ou pas renseignées étaient exclues de l′analyse. Le test t de Student était utilisé pour la comparaison des moyennes lorsque les variances étaient homogènes. Le test du chi^2^ ou le test exact de Fischer ont permis de comparer les proportions des variables catégorielles. Pour rechercher une association entre la variable dépendante qui était le mode de sortie (vivant ou décès) et un facteur de risque présumé, l′Odds ratio (OR) et l′intervalle de confiance à 95% (IC 95%) étaient calculés. Pour l'analyse multivariée, les variables dont les valeurs de p < 0,05 en analyse univariée étaient sélectionnées. Les tests statistiques donnant des valeurs de p < 0,05 étaient considérés significatifs. Nous avons utilisé la 10e édition de la Classification Internationale des Maladies et des problèmes de santé connexes (CIM-10) pour codifier les diagnostics [7].

### Définitions

Un nombre de consultations prénatales (CPN) inférieur à 4 était jugé faible et normal quand il était égal ou supérieur à 4. Selon leur âge, les mères étaient classées en trois groupes: jeunes (≥ 20 ans), intermédiaires (21-34 ans) et âgées (≤ 35 ans). Un accouchement était intra-muros s′il avait eu lieu au sein de la maternité de la CFS et extra-muros s′il avait lieu en dehors. Les nouveau-nés ont été classés en trois groupes selon le poids de naissance: les eutrophiques (2500-3999 g), les faibles poids de naissance (< 2500 g) et les hypertrophiques (ὄ 4 000 g). Selon la période néonatale, on distinguait la période néonatale précoce (0-6 jours révolus) et la période néonatale tardive (7-28 jours révolus). Nous avons regroupé sous le terme d′infections néonatales les infections spécifiques de la période périnatale (P35-P39) et certaines maladies infectieuses et parasitaires acquises après la naissance (A00-B99). L′anémie néonatale était définie pour un taux d′hémoglobine < 150 g/L et l′hypoglycémie pour une glycémie < 2.0 mmol/L.

## Résultats

Durant ses 10 premières années de fonctionnement, l′UN de la CFS a reçu 697 nouveau-nés en hospitalisation.

### Caractéristiques descriptives des mères des nouveau-nés

L′âge était précisé chez 298 femmes et elles avaient en moyenne 28.3 ± 5.91 ans (extrêmes 16 et 43 ans). Les jeunes mères constituaient 10.7% de l′effectif, les âgées 9.2% et les intermédiaires 70.1%. Le nombre moyen de grossesses par femme était 2.4 ± 1.39 (extrêmes 1 et 6), les primigestes étaient 132/398 (33.1%) et les multigestes 266/398 (66.9%). La parité moyenne était 2.1 ± 1.27 (extrêmes 1 et 7), les multipares étaient 225/401 (56.1%) et les primipares 176/401 (43.9%). Le nombre moyen de CPN était 3.7 ± 1.31 (extrêmes 1 et 8); ces CPN étaient faibles chez 95/217 (43.8%) femmes et normales chez les autres.

### Caractéristiques descriptives des nouveau-nés

Les nouveau-nés étaient âgés en moyenne de 2.0 ± 4.6 jours à leur admission, ceux qui âgés de 0-6 jours étaient 640/696 (92.0%) et les 7-28 jours 56/696 (8.0%). Il y avait 361/653 (55.3%) garçons, 290/653 (44.4%) filles et 2/653 (0.3%) de sexe indéterminé, soit un sex ratio de 1.2. L′âge moyen gestationnel était 37.4 ± 3.53 SA (extrêmes 25 et 43 SA); les nouveau-nés à terme étaient 368/574 (64.1%), les prématurés 193/574 (33.6%) et les post-termes 13/574 (2.3%). Le poids moyen de naissance était 2632 ± 753,44 g (extrêmes 600-5100 g). Il y avait 352/619 (56.9%) nouveau-nés eutrophiques, 249/619 (40.2%) faibles poids de naissance et 18/619 (2.9%) hypertrophiques. Dans 403/522 (77.2%) des cas, les nouveau-nés étaient nés intra-muros, les autres (22.8%) étaient nés extra-muros. Les nouveau-nés étaient nés par voie basse dans 355/620 (57.2%) des cas et par césarienne dans 42.8% des cas. Il y avait une rupture prématurée des membranes avant l′accouchement chez 100/187 (53.5%) nouveau-nés. A leur admission, la température moyenne était 37.2 °C ± 1,4 (extrêmes 30.2 et 4.8 °C). Ils étaient admis dans l′UN par transfert direct de la maternité (72.3%), par transfert des urgences pédiatriques (20.4%), par décision du pédiatre après une consultation ordinaire (7.3%) des cas.

### Morbidité

**Figure 1:**
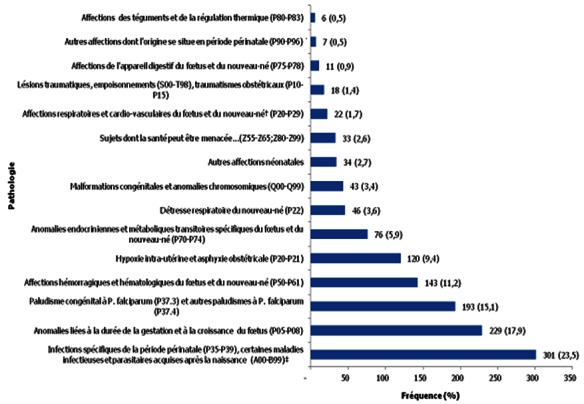
Les pathologies diagnostiquées chez les nouveau-nés hospitalisés entre 1999 et 2009 à Ouagadougou, Burkina Faso (n = 128)

Les infections néonatales étaient les affections les plus fréquentes (301 cas; 23.5% des diagnostics). Elles étaient suivies des anomalies liées à la durée de la gestation et à la croissance du fætus (229 cas; 17.8%) parmi lesquelles il y avait 193 prématurés (33.6% des 574 nouveau-nés dont l′âge gestationnel était connu). Il y avait 193 (15.1%) cas de paludisme, tous dus à *Plasmodium falciparum*, dont 170 cas de paludisme congénital-maladie et 23 cas de paludisme néonatal. Les affections hémorragiques et hématologiques du fætus et du nouveau-né étaient la quatrième pathologie la plus fréquente (143 cas; 11.2%) avec 101 (7.9%) cas d′anémie et 42 (3.3%) cas de syndromes hémorragiques. L′hypoxie intra-utérine et l′asphyxie obstétricale (120 cas; 9.4%) complétait le tableau des cinq premières pathologies néonatales rencontrées dans notre UN. Les anomalies endocriniennes et métaboliques transitoires spécifiques du fætus et du nouveau-né (76 cas; 5.9%) étaient dominées par l′hypoglycémie néonatale (57 cas; 4.4%). La Figure 1 illustre la fréquence des pathologies néonatales rencontrées dans notre étude.

### Evolution intra-hospitalière et mortalité

Après 5.25 ± 8.43 jours d'hospitalisation en moyenne (extrêmes 0 jour et 92 jours), 574/695 (82.6%) nouveau-nés étaient sortis de manière normale, 91 (13.1%) étaient décédés et 30 (4.3%) étaient sortis contre avis médical. Le délai moyen de décès était de 3.0 ± 7.62 jours (extrêmes 0 jour et 67 jours). Les principales causes de décès étaient les anomalies liées à la durée de la gestation et à la croissance du fætus (46.1%), l′hypoxie intra-utérine et l′asphyxie obstétricale (20.9%) et les infections néonatales (17.6%). Les affections les plus létales étaient l′hypoxie intra-utérine et l′asphyxie obstétricale (24.0%) et les anomalies liées à la durée de la gestation et à la croissance du fætus (20.0%). Le Tableau 1 indique les causes de décès et leur mortalité et létalité chez les nouveau-nés dans notre étude. Le décès survenait en période néonatale précoce dans 85/91 (93.4%) des cas et en période néonatale tardive dans les 6 (6.6%) autres cas; le taux de mortalité était de 25.3% (23/91) à J0 d'hospitalisation, de 33.0% (30/91) à J1 et de 81.3% (74/91) entre J0 et J3. Le Tableau 2 présente la mortalité néonatale selon la cause, la tranche d′âge et le délai de survenue du décès.

**Tableau 1:** Les causes de décès et leur mortalité et létalité chez les nouveau-nés hospitalisés entre 1999 et 2009 à Ouagadougou, Burkina Faso

Cause du décès (code CIM-10)	Nombre de cas*	Nombre de décès	Mortalité (%)	Létalité (%)
Anomalies liées à la durée de la gestation et à la croissance du fætus (P05-P08)	210	42	46,1	20,0
Hypoxie et asphyxie obstétricale (P20-P21)	79	19	20,9	24,0
Infections spécifiques période périnatale (P35-P39) et certaines maladies infectieuses et parasitaires acquises après la naissance (A00-B99)	188	16	17,6	8,5
Paludisme congénital à *P. falciparum* (P37.3) et autres paludismes à *P. falciparum* (P37.4)	93	3	3,2	3,2
Détresse respiratoire du nouveau-né (P22)	33	2	2,2	6,1
Malformations congénitales et anomalies chromosomiques (Q00-Q99)	13	2	2,2	15,4
Lésions traumatiques, empoisonnements et autres conséquences de causes externes (S00-T98)	11	2	2,2	18,2
Affections de l'appareil digestif du fætus et du nouveau-né (P75-P78)	9	2	2,2	22,2
Affections hémorragiques et hématologiques du fætus et du nouveau-né (P50-P61)	7	2	2,2	28,6
Anomalies endocriniennes et métaboliques transitoires spécifiques du fætus et du nouveau-né (P70-P74)	7	1	1,1	14,3

* seul le diagnostic principal de sortie a été pris en compte. Effectif total des nouveau-nés hospitalisés = 697. Nombre total de décès = 91.

**Tableau 2:** La mortalité selon la cause, la tranche d'âge et le délai de survenue du décès chez les nouveau-nés hospitalisés entre 1999 et 2009 à Ouagadougou, Burkina Faso (n = 91)

Causes du décès (code CIM-10)	Nombre de décès par tranche d'âge (%)			Délai moyen de décès (jours)
	J0-J6	J7-J28	Total	
Anomalies liées à la durée de la gestation et à la croissance du fætus (P05-P08)	42 (46,1)	0 (0,0)	42 (46,1)	4,4
Hypoxie intra-utérine, asphyxie obstétricale (P20-P21)	19 (20,9)	0 (0,0)	19 (20,9)	4,5
Infections spécifiques de la période périnatale (P35-P39) et certaines maladies infectieuses et parasitaires acquises après la naissance (A00-B99)	13 (14,3)	3 (3,3)	16 (17,6)	1,4
Paludisme congénital à *P. falciparum* (P37.3) et autres paludismes à *P. falciparum* (P37.4)	2 (2,2)	1 (1,1)	3 (3,3)	1,7
Détresse respiratoire du nouveau-né (P22)	2 (2,2)	0 (0,0)	2 (2,2)	1,5
Malformations congénitales et anomalies chromosomiques (Q00-Q99)	2 (2,2)	0 (0,0)	2 (2,2)	0
Lésions traumatiques, empoisonnements et certaines autres conséquences de causes externes (S00-T98)	2 (2,2)	0 (0,0)	2 (2,2)	1,5
Affections de l'appareil digestif du fætus et du nouveau-né (P75-P78)	1 (1,1)	1 (1,1)	2 (2,2)	9,0
Affections hémorragiques et hématologiques du fætus et du nouveau-né (P50-P61)	2 (2,2)	0 (0,0)	2 (2,2)	0,5
Anomalies endocriniennes et métaboliques transitoires spécifiques du fætus et du nouveau-né (P70-P74)	0 (0,0)	1 (1,1)	1 (1,1)	0,5

### Les facteurs de risque de décès néonatal

En analyse univariée, six des 11 facteurs de risque étudiés était statistiquement associés au décès du nouveau-né. C’était la primigestité (OR = 1.77; IC 95% (1.07-2.95); p = 0.03), le faible nombre de CPN (OR = 3.44; IC 95%(1.26-9.35); p = 0.01), l'accouchement extra-muros (OR = 3.09; IC 95% (1.70-5.60); p = 0.000), la voie basse d′accouchement (OR = 2.64; IC 95% (1.55-4.50); p = 0,000), la prématurité (OR = 2.42; IC 95% (1.54-3.82); p = 0,000), et le faible poids de naissance (OR = 2.10; IC 95% (1.31-3.37); p = 0.000). En analyse multivariée, un seul des six facteurs qui étaient significatifs en analyse univariée était demeuré significatif. C′était la voie basse d′accouchement (OR = 6.57; IC 95% (1.33-32.29); p = 0.02) (Tableau 3).

**Tableau 3:** Les facteurs de risque de décès des nouveau-nés hospitalisés entre 1999 et 2009 à Ouagadougou, Burkina Faso

Facteur de risque de décès	Analyse univariée			Analyse multivariée		
	OR	[IC 95%]	*p*	OR	[IC 95%]	*p*
Jeune âge de la mère	0,45	[0,10-1,96]	0,39			
Primigestité	1,77	[1,07-2,95]	0,03	2,08	[0,67-6,45]	0,20
Primiparité	1,45	[0,90-2,34]	0,13			
Faible nombre de CPN	3,44	[1,26-9,35]	0,01	2,03	[0,61-6,71]	0,24
Pathologie sur grossesse	2,43	[0,83-7,15]	0,10			
RPM*	1,11	[0,50-2,45]	0,79			
Accouchement extra-muros	3,09	[1,70-5,60]	0,000	1,86	[0,37-9,30]	0,45
Voie basse d'accouchement	2,64	[1,55-4,50]	0,000	6,57	[1,33-32,29]	0,02
Sexe masculin	1,00	[0,63-1,58]	0,99			
Prématurité	2,42	[1,54-3,82]	0,000	3,58	0,61-20,95	0,16
Faible poids de naissance	2,10	[1,31-3,37]	0,002	2,27	[0,39-13,19]	0,36

* Rupture Prématurée des Membranes avant l'accouchement. OR: Odd-ratio. [IC 95%]: intervalle de confiance à 95%. Pour l'analyse multivariée, seules les variables dont les valeurs de *p* < 0,05 en analyse univariée étaient sélectionnées

## Discussion

Le caractère rétrospectif de l′étude n′a pas permis un recueil exhaustif de toutes les données par manque de complétude de certains dossiers cliniques. En outre, l′UN de la CFS n′accueille pas tous les nouveau-nés non seulement à cause de sa petite capacité mais aussi à cause de son statut privé qui la rend inaccessible financièrement aux populations à faibles revenus ou n′ayant pas une Assurance-maladie. Malgré ces limites, la longue période d′étude nous a permis d′obtenir un échantillon de grande taille pour l′analyse et l′interprétation des données sur la morbidité et la mortalité des nouveau-nés.

La liste des pathologies du nouveau-né est pratiquement la même en Afrique sub-saharienne où seul leur ordre de fréquence varie selon les études. Il s′agit principalement des infections (35,9 à 73.9%), de la prématurité (15.9 à 29.9%), de l′asphyxie (13.5 à 41.5%), de la détresse respiratoire (3.5 à 15.6%) [8–13]. Sont moins fréquents l′anémie (3.8%) et les syndromes hémorragiques (3.1%) [8], les intoxications aiguës accidentelles (4.4%) [10] et les syndromes malformatifs (4.3% à 8.8%) [9–11]. Sous nos latitudes, les infections néonatales bactériennes sont très souvent diagnostiquées sur des arguments cliniques et/ou biologiques [10–12], leur preuve bactériologique étant rarement faite alors que celle-ci est indispensable pour un traitement antibiotique rationnel efficace [14, 15]. L′origine beaucoup plus communautaire que materno-faetale des germes en cause traduit un défaut d′hygiène à l′accouchement dans les maternités des pays en développement [16] et dans les services de néonatologie où l′ampleur des infections nosocomiales mérite d′être précisée. Le dépistage et le traitement précoces des infections maternelles (surtout génito-urinaires), le respect des règles élémentaires d′asepsie (en particulier les soins du cordon ombilical) à l′accouchement sont des mesures de prévention qui, associées à la prise en charge précoce des cas permettraient de réduire de 38 à 78% les infections néonatales ou encore de sauver 47 à 82% des vies des nouveau-nés [3, 4]. Dans notre étude, une importante proportion de femmes n′avait pas suivi le nombre recommandé de quatre CPN. Or celles-ci sont des moments privilégiés pour mener ces activités de prévention des infections néonatales. Ces occasions manquées expliqueraient en partie l′incidence élevée des infections néonatales que nous avons observées. La lutte contre l′accouchement prématuré est un objectif majeur des CPN mais nous avons observé un taux élevé de nouveau-nés prématurés dans notre étude. Plus que le déficit en nombre, l′on peut aussi se poser des questions quant à la qualité même des CPN. Par exemple, nous avons observé que le paludisme était fréquent au point d′occuper la 3e place des affections néonatales dans notre étude. Pourtant, les mesures individuelles et collectives de prévention doivent être rappelées et vérifiées au besoin à l′occasion afin de prévenir le paludisme de la femme enceinte dont les conséquences sont préjudiciables au fætus et au nouveau-né: avortements, mort-nés, faible poids de naissance, prématurité [17], paludisme congénital [18].

Malgré les progrès enregistrés [19], la mortalité néonatale demeure préoccupante en Afrique où un quart à plus d′un tiers des nouveau-nés meurt selon les données hospitalières [8, 9, 11, 12, 20]. Elle paraît cependant moins élevée au Burkina où le taux trouvé dans notre étude (13.1%) était encore en deçà des 15.3% et 25.9% rapportés par les études antérieures menées, il est vrai, dans des hôpitaux publics du pays [10, 13]. Au Nigéria, le taux de mortalité néonatale est aussi moins élevé et varie entre 4,3 et 20,3% [15, 21–24]. La mortalité néonatale serait un indicateur de développement d'une collectivité et le reflet de la qualité des soins obstétricaux et néonatals dispensés dans un établissement de santé [20]. C′est en période néonatale précoce que les chiffres de mortalité sont encore plus dramatiques. En effet, les taux de mortalité dans cette période culminent à plus de 80% dans les pays africains [8, 9, 11–13, 20]. Dans un ordre ou dans un autre, ce sont toujours les mêmes causes de mortalité qui sont rapportées en Afrique, à savoir les infections néonatales (13,9 à 30,3%), l′asphyxie à la naissance (10,8 à 50%) et la prématurité et ses complications (25,3 à 42,9%) [9, 11–13, 15, 22, 24]. Ces données concordent avec les résultats d′une étude portant sur 7 993 grossesses dans six pays en développement qui montraient que la prématurité était la principale cause de décès dans 62% des cas [25]. Ces trois causes directes sus citées sont connues pour être responsables à elles seules de 86% des décès néonatals de part le Monde [1]. Elles ne sont en fait que la partie visible d′un iceberg dont la partie immergée cache mal de nombreuses causes constitutives ou sous-jacentes aux niveaux du ménage, de la communauté et du district, elles-mêmes soutenues par des causes de base au niveau sociétal. Les nombreux décès néonatals enregistrés sont donc le résultat de la conjonction de toutes ces causes et facteurs [26]. Ces chiffres dramatiques renvoient une fois de plus aux problèmes de la qualité des soins à l′accouchement, de la prise en charge du nouveau-né en général et du prématuré en particulier dans notre contexte de pays en développement. Ils montrent également l′ampleur du drame, la difficulté du chemin à parcourir et l′immensité des efforts à fournir pour atteindre les OMD liés à la santé maternelle et infantile.

Dans notre étude, la voie basse d′accouchement était la voie prépondérante d′accouchement mais aussi un facteur de risque de décès néonatal. Cette voie semble exposer à plus de risque de dystocie, source d′hypoxie intra-utérine et d′asphyxie à la naissance et donc de décès si des soins adéquats de réanimation néonatale ne sont pas appliqués. Ceci expliquerait pourquoi l′hypoxie anténatale et l′asphyxie obstétricale constituaient l′une des principales causes de mortalité néonatale observée dans notre étude. On ne peut que rappeler ici l′importance de l′utilisation du partogramme dans le monitorage de l′accouchement. En cas d′anomalie de progression du travail, il donne l′alerte à l′équipe obstétricale qui doit prendre une prompte décision telle qu′une césarienne afin d′extraire le fætus de l′atmosphère délétère d′une souffrance [27]. La césarienne semblait être un facteur protecteur de décès néonatal dans notre étude. On pense même qu′elle permettrait d′éviter 71% des morts périnatals [3]. Pour prévenir l′asphyxie néonatale, des soins prénataux de qualité permettant de dépister les situations à risque et une assistance à l′accouchement par du personnel qualifié sont fondamentaux. Il y a aussi la mise en place de soins obstétricaux et néonataux d′urgence subventionnés qui contribuera à rendre les services de santé plus accessibles à la grande majorité pauvre des populations de nos pays en développement afin de réduire la mortalité néonatale (et maternelle). On estime que l′application de telles mesures permettrait de sauver la vie de 39 à 71% de nouveau-nés [4].

## Conclusion

Le bilan de la mortalité néonatale est lourd dans les pays en développement où les nouveau-nés continuent de mourir pour des causes souvent évitables. Des consultations prénatales en nombre recommandé et de bonne qualité, un renforcement de la surveillance des accouchements par voie basse par l'utilisation du partogramme, un élargissement des indications de la césarienne, et une prise en charge efficace du nouveau-né dans sa première semaine de vie, surtout dans ses trois premiers jours, amélioreraient le pronostic néonatal.
